# A Multi-Method Approach to Curriculum Development for In-Service Training in China’s Newly Established Health Emergency Response Offices

**DOI:** 10.1371/journal.pone.0100892

**Published:** 2014-06-27

**Authors:** Yadong Wang, Xiangrui Li, Yiwen Yuan, Mahomed S. Patel

**Affiliations:** 1 School of Health Management and Education, Capital Medical University, Beijing, China; 2 Beijing Cancer Hospital, Beijing, China; 3 Peking University Third Hospital, Beijing, China; 4 National Centre for Epidemiology and Population Health, Australian National University, Canberra, Australia; Curtin University, Australia

## Abstract

**Objective:**

To describe an innovative approach for developing and implementing an in-service curriculum in China for staff of the newly established health emergency response offices (HEROs), and that is generalisable to other settings.

**Methods:**

The multi-method training needs assessment included reviews of the competency domains needed to implement the International Health Regulations (2005) as well as China’s policies and emergency regulations. The review, iterative interviews and workshops with experts in government, academia, the military, and with HERO staff were reviewed critically by an expert technical advisory panel.

**Findings:**

Over 1600 participants contributed to curriculum development. Of the 18 competency domains identified as essential for HERO staff, nine were developed into priority in-service training modules to be conducted over 2.5 weeks. Experts from academia and experienced practitioners prepared and delivered each module through lectures followed by interactive problem-solving exercises and desktop simulations to help trainees apply, experiment with, and consolidate newly acquired knowledge and skills.

**Conclusion:**

This study adds to the emerging literature on China’s enduring efforts to strengthen its emergency response capabilities since the outbreak of SARS in 2003. The multi-method approach to curriculum development in partnership with senior policy-makers, researchers, and experienced practitioners can be applied in other settings to ensure training is responsive and customized to local needs, resources and priorities. Ongoing curriculum development should reflect international standards and be coupled with the development of appropriate performance support systems at the workplace for motivating staff to apply their newly acquired knowledge and skills effectively and creatively.

## Introduction

The outbreak of severe acute respiratory syndrome (SARS) in 2003 was a watershed moment for China [1]. It triggered major health reforms [1]–[4] and led to an increase in public health funding of about 100% by 2007, accounting for a rise in spending from 0.75% to 0.89% of the gross domestic product [1]. Subsequent emergencies that echoed the imperative for reforms included the outbreaks of influenza H5N1 in birds and humans [1]–[4], melamine contamination of milk formula that affected over 294,000 Chinese children [5], and the earthquake in Sichuan that resulted in over 69 000 deaths, displaced about 15 million people and led to the mobilization of over 10 000 medical workers [6].

The reforms to strengthen China’s emergency preparedness and response capabilities included new laws and regulations [7]–[10], increased support for training and research, and the adoption of international best practice [2]–[4], [11]. To help implement the new laws, Health Emergency Response Offices (HERO) were established within China’s health administration units at the national, provincial and municipal levels, in Centers for Disease Control (CDC) and in tertiary hospitals [12]. The HEROs within any one province now collectively employ around 1000 staff members, and their role is to help develop and coordinate preparedness planning and emergency response within their jurisdictions, as well as implement the International Health Regulations (2005) (IHR) [13]. However, because staff members were recruited opportunistically from diverse professional backgrounds into the newly established HEROs, they had not been trained systematically in emergency preparedness and response previously. While they received in-service training, this was offered in an *ad hoc* manner, was not preceded by a needs assessment [14], and was aimed primarily at improving knowledge of the new laws that did not translate directly into strengthening performance of the HEROs [15], [16].

At the global level, perennial threats of the pandemic spread of infectious diseases like SARS and influenza, as well as the sequelae of earthquakes, tsunamis, bioterrorism and complex humanitarian emergencies, heightened awareness of the need to strengthen national, regional and global capacity in prevention, preparedness and response to public health emergencies. In 2004, the World Association for Disaster and Emergency Medicine (WADEM) proposed a framework for disaster health to facilitate the development of educational programs in the field [17]. Efforts were directed at identifying and defining criteria for assessing disaster health-related competencies, standards for guiding curricular development, and exploring the methods, duration and desired outcomes of training [17]–[19]. Australia for example, developed a national framework for disaster health education to provide guidance for educators to achieve a standardized and integrated approach across the country [20]. Australia adapted the WADEM recommendations to target seven educational levels, and outlined the curricular contents and outcomes for each level; the seven levels were defined as 1) informing the community, 2) raising awareness of health workers, 3) providing basic knowledge and skills for health workers, 4) advancing the knowledge of health workers who play lead roles in disaster management, 5) enhancing expert knowledge among a small group of health workers, 6) targeting specialist level amongst a small group of individuals, and 7) encouraging research and innovation to further develop the knowledge base of disaster health [20].

In recognition of the national need for an in-service program to target the “level four” health staff as defined by WADEM, i.e. “health workers who played lead roles in disaster health management” [20], China’s Ministry of Health (MoH) commissioned the Capital Medical University in 2010 to develop and implement a competency-based curriculum to help strengthen the performance of the new cadre of HERO staff. An immediate priority was to address the WHO requirements for countries to meet the core capacity requirements for implementing the IHR by June 2012 [21]. This paper outlines the consultative process used to conduct the training needs assessment and develop the curriculum for implementation across China, with support from WHO. The multi-method approach to curriculum development can be applied in other settings to ensure training is responsive and customized to local needs, resources and priorities.

## Methods

### Ethics Statement

We did not submit the study proposal for ethics approval because we conducted meetings and interviews with study informants on a voluntary basis for the sole purpose of identifying training needs at the workplace; we did not gather any personal information or attributes about individual informants beyond their age and past work experience.

**Figure 1:**
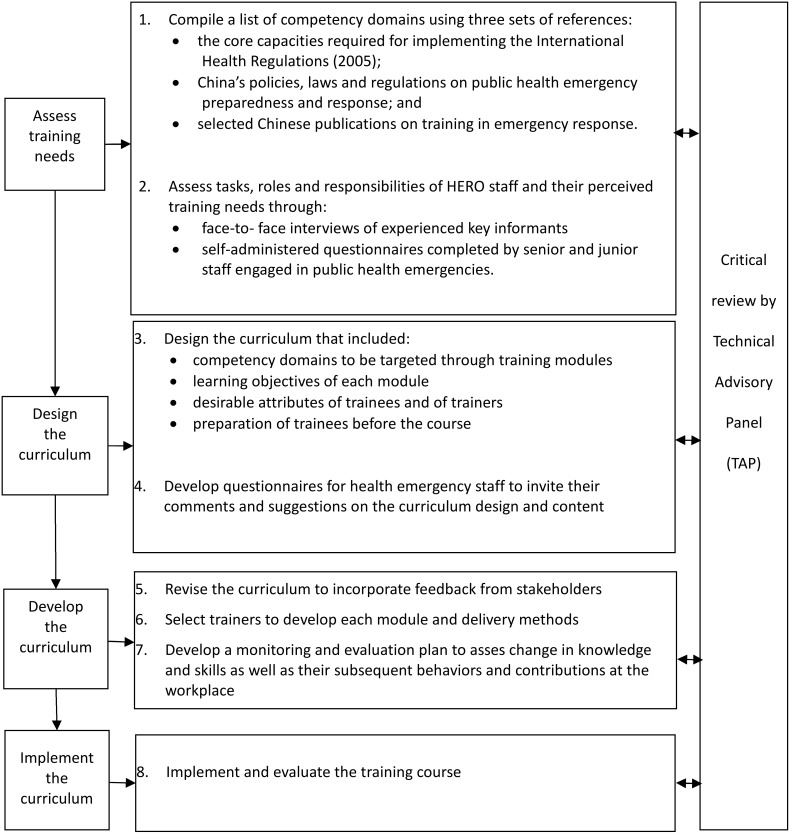
Adaptation of the ADDIE model to develop the national in-service curriculum for strengthening the performance of staff of health emergency response offices (HEROs).

Figure 1 outlines the approach for developing the in-service curriculum using ADDIE as the basic framework for instructional design [22]. Each of the five phases of this model – Assessment, Design, Development, Implementation, and Evaluation – were reviewed critically by a technical advisory panel (TAP). The panel offered suggestions for strengthening the curriculum development process, as well as the content, relevance and quality of the curriculum. The multi-method training needs assessment and subsequent steps of the ADDIE model are detailed below.

### Assessment of training needs

We defined a competency as “a cluster or related knowledge, skills, and attitudes that reflects a major portion of one’s job (a role or responsibility), that correlates with performance on the job, that can be measured with well-accepted standards, and that can be improved with training and development” [23]. A competency domain incorporated an inter-related group of competencies for performing specified tasks.

In order to identify the list of competency domains against which training needs of HERO staff could be assessed, we collated data from three sets of references. The first was from the IHR core capacities necessary to detect, assess, report and respond to emergencies [21]. The second set of references was from inter-related Chinese laws and regulations: the *Emergency Response Law* which is the legal framework for managing emergencies [7], the *National Health Emergency Training Outline* which identifies the knowledge and skills to be targeted by training programs [11], and the *Regulations on Preparedness for and Response to Emerging Public Health Hazards* which outlines preparedness and response activities [8], [10]. The final set of references was selected from Chinese publications on general guidelines for training health workers [24] and from curricula for training technical staff based in disease surveillance units, laboratories and the environmental health sector [14]–[16], [24]–[27].

We assessed tasks, roles and responsibilities, and training needs of HERO staff through face-to- face interviews of eleven experienced key informants; they included health emergency experts from the government, the military and the academic sector, and senior staff of HEROs. We also explored their awareness of existing training activities and the associated relative strengths and weaknesses, their preferences on modes of curriculum delivery and the optimal duration of in-service training. We collected data on the same issues through self-administered questionnaires to a group of 115 HERO staff members from across China when they attended a training workshop in Beijing.

China’s Ministry of Health (MoH) appointed a technical advisory panel (TAP) to critically review and offer comments and suggestions on all aspects of the needs assessment and curriculum development process, as well as on the relevance and quality of the curriculum. The eight panel members were nationally acknowledged experts in public health emergencies from the MoH, CDCs, HEROs and the Academy of Military Medical Science.

### Designing and developing the curriculum

We used the results of the needs assessment to design the draft curriculum that included the topics listed in Figure 1. Each of the identified competency domains was targeted through a self-contained module that could be run independently of the other modules. Each module encompassed theories, laws, concepts and tools and techniques essential for HERO staff to perform effectively in their work.

We developed questionnaires for HERO staff to invite their comments and suggestions on the curriculum design, and the expected level of need and potential demand for the training. The questionnaires were disseminated with the draft curriculum to 700 HERO staff members at the provincial and municipal levels and 1000 health staff responsible for responding to emergencies at the local level. We also invited comments on the curriculum from the Technical Advisory Panel and staff of the WHO Office in China. We used their feedback to revise the draft curriculum.

### Implementing and evaluating the curriculum

The revised curriculum was endorsed by the Technical Advisory Panel and the Ministry of Health before it was implemented, monitored and evaluated between June and November 2010.

The key tasks of HERO staff in their respective jurisdictions were identified as coordination of the development and implementation of preparedness plans and early warning surveillance systems, mobilization of emergency response teams, and implementation of the IHR [13]. The list of competency domains associated with these tasks is shown in Table 1. Domains 1 to 8 were transcribed from both the IHR [13], [21] and the Chinese references [7], [10], [11]. Domains 9 to 18 were explicit only in the latter set of references; although implicit in the IHR capacities, study respondents suggested they be considered as discrete competency domains.

**Table 1:** List of competency domains to be considered for inclusion in the curriculum.

Competency domains	Reference
**Domains defined as the core capacities for implementing the IHR**	
1. National legislation, policy and financing	[10], [21]
2. Coordination and National Focal Point communications	[7], [21]
3. Surveillance	[7], [10], [21]
4. Response	[7], [10], [11], [21]
5. Preparedness	[7], [10], [11], [21]
6. Risk communications	[7], [10], [11], [21]
7. Human resources	[7], [10], [11], [21]
8. Laboratory	[10], [11], [21]
**Domains not explicit in the IHR core capacities**	
9. Health promotion and community education	[7]
10. Resource management and stockpiling	[7], [10], [11]
11. Risk assessment and management	[7], [10], [11]
12. Monitoring compliance with laws and regulations	[7], [10]
13. Decision-making processes for alerting the community	[7], [10]
14. Medical rescue	[10]
15. On-site organization during response	[7], [10]
16. Managing recovery after an emergency	[7], [10], [11]
17. Evaluation of response to emergencies	[7], [10]
18. Research on emergencies	[7]

### Training needs assessment

Of the 1700 health professionals to whom questionnaires were distributed, 1606 (94.5%) responded with comments and suggestions; selected characteristics of these respondents are summarized in Table 2. Of the respondents, just under two-thirds were aged 40 years and over, 45% had worked previously as clinical practitioners, 35% as preventive health (public health) practitioners, and 8% as health administrators. Overall, 58% had less than 5 years experience in emergency response work.

**Table 2:** Selected characteristics of the 1606 health staff who responded to the curriculum development questionnaire.

Characteristics of respondents		County level n = 912 (%)	Municipal level n = 551 (%)	Province level 143 (%)	Total 1606 (%)
**Age (Years)**	19–29	42 (5.1)	31 (6.1)	17 (13.9)	90 (6.2)
	30–39	248 (30.0)	154 (30.3)	36 (29.5)	438 (30.1)
	40–49	451 (54.6)	248 (48.8)	58 (47.5)	757 (52)
	50–62	85 (10.3)	75 (14.8)	11 (9.0)	171 (11.7)
**Past work experience**	Clinical medicine	395 (44.9)	254 (47.6)	47 (36.4)	696 (45.1)
	Preventive medicine	322 (36.6)	174 (32.6)	42 (32.6)	538 (34.9)
	Health administration	64 (7.3)	39 (7.3)	21 (16.3)	124 (8)
	Other	99 (11.3)	67 (12.5)	19 (14.7)	185 (12)
**Workplace**	Administrative unit	99 (11.3)	67 (12.5)	19 (14.7)	185 (12)
	Hospital	235 (26.6)	189 (35.2)	68 (51.5)	492 (31.7)
	CDC	244 (27.6)	124 (23.1)	33 (25.0)	401 (25.8)
**Past experience in emergency response**	0–5 years	477 (57.9)	289 (58.7)	74 (58.7)	840 (58.3)
	6–10 years	202 (24.5)	119 (24.2)	31 (24.6)	352 (24.4)
	>10 years	145 (17.6)	84 (17.1)	21 (16.7)	250 (17.3)

Note: Not all characteristics of the 1606 respondents at various levels were known.

The major weaknesses the respondents, key informants and the Technical Advisory Panel identified in existing training programs were the didactic methods used for classroom teaching and the emphasis on hazard-specific approaches (such as for responding to SARS or pandemic influenza or earthquakes), and on acquiring knowledge of the new laws and regulations rather than on addressing the challenges to their implementation. Furthermore, while training programs in the past had targeted knowledge and skills required by technical staff such as epidemiologists, laboratory scientists and environmental staff, they had not incorporated skills in mobilizing, managing and coordinating teams across multiple sectors, disciplines and agencies. The respondents stressed the need for adopting interactive learning methods and problem-solving exercises in the classroom, as had been suggested in Chinese publications [26]–[29]. They supported replacing the ‘hazard-specific’ approach with the ‘all hazards’ approach to managing emergencies, development of a competency-based curriculum with modules that addressed specific sets of competency domains, and limiting the duration of in-service training to about 2 to 3 weeks so these interfered minimally with trainees’ regular work responsibilities.

### Designing the draft curriculum

After discussions with the key informants and TAP, 11 of the 18 competency domains shown in Table 1 were to be developed into the individual modules shown in Table 3. They included six of the eight IHR-related competencies, while the remaining two, ‘national legislation’ and ‘national co-ordination’ were omitted because they were directed mainly at national-level decision-makers. The other five domains were specified in the Chinese references, namely on-site organization during response, decision-making processes for alerting the community, resource management and stockpiling, risk assessment and management, and evaluation of response to emergencies. The remaining seven domains shown in Table 1 were not developed into modules because they were considered to be a lower priority for health managers and could not be included in the first round of in-service training to be conducted over a limited 2–3 week timeframe.

**Table 3:** Proportion of HERO staff who expressed a high level of need for the eleven modules.

Competency domain and related modules	High Level of need (%)
1. On-site organization during response	92
2. Preparedness	91
3. Decision-making processes for alerting the community	87
4. Surveillance	84
5. Resource management and stockpiling	84
6. Risk assessment and management	84
7. Human resources	83
8. Response	80
9. Evaluation of response	72
10. Risk communication	71
11. Laboratory capacity	57

The proportion of the 1606 respondents who expressed a high level of need for these modules are shown in Table 3.

Case studies and desktop simulation exercise were both ranked as the preferred training method by 94% of respondents, followed by lectures (85%) and case studies (85%). The median preferred time for each module was 2.5 days. To accommodate this request and complete the course within 3 weeks, we included only nine modules, and excluded modules for two domains considered not to be priorities for HEROs: human resources and laboratory.

### Developing the curriculum

The curriculum was revised to incorporate feedback from the stakeholders. Two trainers prepared each module to be run over 2 to 2.5 days; one was typically a university academic with nationally acknowledged expertise in emergencies, and the other, a senior staff member of a HERO. Each module would be delivered through three sessions: a lecture on relevant theories, concepts and tools and techniques that were followed by a case study and a desktop simulation exercise; the latter two sessions were conducted with small groups of 6 to 8 participants as an opportunity to apply, experiment with, and consolidate the knowledge acquired from the lectures.

Before the course, trainees were to be provided with readings for each module, and invited to prepare appropriate material from their workplace for a case study relevant to at least one module; this could include a preparedness plan, report on a risk assessment, a risk communication plan, or other reports on an emergency event. The trainers then prepared case studies either from the participants’ material, or designed them *de novo* based on their own experiences. An example of the former was a case study based on a jurisdictional preparedness plan and where participants had to identify strengths, weaknesses and contentious issues, and to explore alternative options in the light of the newly acquired knowledge from the lectures. On returning home, they were expected to review and revise their jurisdictional plan using a similar learning approach with their local team members. An example of a case study designed by the facilitator was the assessment and management of the risk of an infectious disease outbreak at a conference attended by about 1000 international participants. Each small group would then be expected to present a 20 minute report for further discussions at a plenary, with a synthesis of key learning points.

An example of a desktop simulation exercise developed by the facilitator was an earthquake scenario where participants had to convene a national response team, to mobilize them within a safe operational base near the affected zone, and then arrange the logistics for transporting and storing essential medical supplies and equipment. Each small group would prepare and submit written responses to the evolving scenario to which they would receive feedback from the facilitators.

The delivery of the modules was to be monitored to document attendance of trainers and trainees, the content of the training materials and the mode of delivery of each module. The evaluation plan was derived from the four Kirkpatrick levels of evaluation [30]; the first two levels, ‘reaction’ to the learning content and environment and ‘learning’ (or acquisition of new knowledge) were both assessed immediately after each module. The next two levels, ‘behavior’ at the workplace and ‘results’ (or the benefits of change in behavior at the workplace) were assessed three months later.

The results of the needs assessment and curriculum were presented, debated and finalized at a workshop with key informants, TAP members and senior HERO staff.

### Implementing and evaluating the curriculum

The first in-service course was delivered to a class of 37 HERO staff members from 31 provinces across China; each participant had at least one undergraduate degree and over two-year’s work experience in emergency response. The monitoring and evaluations activities were implemented as planned, and the results will be published separately.

## Discussion

This study on developing an in-service curriculum for new professional cadre of HERO staff from across the country adds to the literature on China’s health reforms and enduring efforts to strengthen emergency response capabilities following on the SARS outbreak and other emergencies [1]–[4]. It describes the multi-method approach used to identify training needs systematically, and to adopt international best practice in partnership with senior decision-makers and content experts from the government, academia and the military. The study revealed the overwhelming need to replace didactic teaching, the classical method used across China, with active learning and training methods. The consultative process for developing the curriculum was designed to address the scale of the challenge for coordinating planning and training activities across jurisdictions that cover a population of over 1.3 billion people, and where most provinces have more than 60 million residents. The process required active engagement with experts in government, academia and the military, as well as inputs from 1606 immediate potential beneficiaries. The expert technical advisory panel played a critical role through each step in the ADDIE model, and endorsed the curriculum development process as well as the contents of the curriculum.

The IHR (2005) core capacities are aimed at minimizing the international impact of communicable disease emergencies and chemical, radiation and nuclear accidents [13], [21]. However, they are also essential for timely and effective detection of, and responses to, the emerging threat at its source. China’s reforms to strengthen preparedness and response capabilities [2]–[4], [7]–[11] therefore incorporated the IHR core capacities as well as the competency domains identified as priorities based on China’s recent experiences with emergencies [1]–[6].

The overwhelming rejection of didactic teaching methods by the study respondents and the need to replace these with active learning and teaching methods was encouraging news for the curriculum designers. This approach is consistent with the two Chinese symbols that depict “learning” (??: xué xí) - ‘acquisition of knowledge’ and ‘repeated practice’ as inseparable sides of the one coin. Training aimed at strengthening performance has to create opportunities for active learning and experimentation with problem solving exercises to apply and consolidate newly acquired knowledge and skills.

The curriculum marks the beginning of a new journey for strengthening the performance of the recently established HEROs in jurisdictions across China. This initial effort focused strongly on meeting the WHO requirements for developing the core capacities required to implement the IHR [21], and in particular, to strengthen the competencies of the “level four” health staff [20] who play lead roles in emergency planning and management. Our approach for identifying and developing competencies and standards are consistent with those published by WADEM in 2013 [18], [19], and in many ways resemble those used in Australia [20]. The initial list of 18 competency domains were derived from the eight core capacities required to implement the IHR [21] and the disaster management cycle of prevention, preparedness, response and recovery [17], both of which are embedded in Chinese laws and regulations [7]–[12]. The scope of the task and all aspects of the needs assessment and identification of the competencies were developed through iterative inputs from subject matter experts in the Ministry of Health, academia and the military. The mode of curriculum delivery was based on blended learning methods implicit in Bloom’s taxonomy as outlined in the Australian framework [20]. The duration of training for the level four workers In Australia is recommended for 40 hours [20]; by contrast, our course was conducted over 100 hours because the target group had received little training in the past and included workers with diverse sets of past experience. However, consistent with the WADEM guidelines [17]–[20], China needs to reconcile its curriculum with international standards and expand its efforts to develop and standardize training frameworks for the other six WADEM levels [20], as well as interconnect with the “cross-cutting” competencies of workers from the other disciplines that participate in emergency responses [19]. Adopting the international standards and practice will also enhance communications, inter-agency cooperation and inter-operability across China’s national borders.

The major limitation of our training is that it helps develop only the knowledge, skills and attitudes essential for working in the field of emergency preparedness and response. We recognize that training constitutes but one component of a package of activities needed to strengthen the performance of HERO staff. Training must be linked with an appropriate performance support system [31] that motivates staff to apply the newly acquired knowledge and skills, and in this way, to start contributing effectively and creatively to the workplace. The support system includes a congenial work environment; explicit guidelines, supervision as well as feedback on the quality of their performance; appropriate resources such as materials, equipment and computer software; and incentives such as financial and non-financial rewards for good performance as well as opportunities for career development [31].

The multi-method approach to curriculum development by engaging actively with senior policy-makers, researchers, and experienced practitioners can be applied in other country settings to ensure training is responsive and customized to local training needs, resources and priorities.
